# Supplementary material for: MetaQTL: a package of new computational methods for the meta-analysis of QTL mapping experiments
Source: BMC Bioinformatics. 2007 Feb 8;8:49. doi: 10.1186/1471-2105-8-49 (PMC1808479; doi:10.1186/1471-2105-8-49)
Supplement: Additional File 7 — MetaQTL Package : jar file and tutorial. This Zip archive contains both the MetaQTL JAR file and the files of the tutorial. [file 1471-2105-8-49-S7.zip › org.inra.metaqtl/doc/org/metaqtl/MetaQtlModel.html]

MetaQtlModel


|  |  |  |
| --- | --- | --- |
| **Overview** \| **Package** \| **Class** \| **Use** \| **Tree** \| **Deprecated** \| **Index** \| **Help** | |  |
| **PREV CLASS**   **NEXT CLASS** | **FRAMES**    **NO FRAMES**     **All Classes** |  |
| SUMMARY: NESTED \| FIELD \| CONSTR \| METHOD | DETAIL: FIELD \| CONSTR \| METHOD |  |


---


## org.metaqtl Class MetaQtlModel

```
java.lang.Object
  org.metaqtl.MetaQtlModel
```

---

``` public class MetaQtlModel extends java.lang.Object ```

---

| **Field Summary** | |
| --- | --- |
| `java.lang.String[]` | `chromNames`             The chromosomes. |
| `int[][]` | `models`             The number of Qtl per chromosome and per trait. |
| `java.lang.String[][]` | `traitNames`             The trait names per chromosome. |


**Constructor Summary**

`MetaQtlModel()`

`MetaQtlModel(int nchr)`


| **Method Summary** | |
| --- | --- |
| `java.lang.String[]` | `getChromNames()`             Returns the names of the chromosomes. |
| `java.lang.String` | `getCriterion()` |
| `int` | `getModel(int i, int j)` |
| `int` | `getModel(java.lang.String chrom, java.lang.String trait)` |
| `java.lang.String[]` | `getTraitNames(int cidx)` |
| `java.lang.String[]` | `getTraitNames(java.lang.String chrom)` |
| `void` | `setChromName(int cidx, java.lang.String name)` |
| `void` | `setCriterion(java.lang.String critName)`             Set the criterion name which was used to obtain this model |
| `void` | `setModel(int cidx, int[] models)` |
| `void` | `setTraitNames(int cidx, java.lang.String[] traits)` |

**Methods inherited from class java.lang.Object**

`clone, equals, finalize, getClass, hashCode, notify, notifyAll, toString, wait, wait, wait`

**Field Detail**

### chromNames

```
public java.lang.String[] chromNames
```

:   The chromosomes.

---


### traitNames

```
public java.lang.String[][] traitNames
```

:   The trait names per chromosome.

---


### models

```
public int[][] models
```

:   The number of Qtl per chromosome
    and per trait.


**Constructor Detail**

### MetaQtlModel

```
public MetaQtlModel()
```

---


### MetaQtlModel

```
public MetaQtlModel(int nchr)
```


**Method Detail**

### getChromNames

```
public java.lang.String[] getChromNames()
```

:   Returns the names of the chromosomes.

    :   **Parameters:**: `chrom` - **Returns:**

---


### getTraitNames

```
public java.lang.String[] getTraitNames(java.lang.String chrom)
```

:   **Parameters:**: `chrom` - **Returns:**

---


### getTraitNames

```
public java.lang.String[] getTraitNames(int cidx)
```

:   **Parameters:**: `chrom` - **Returns:**

---


### getModel

```
public int getModel(java.lang.String chrom,
                    java.lang.String trait)
```

:   **Parameters:**: `chrom` -: `string` - **Returns:**

---


### setModel

```
public void setModel(int cidx,
                     int[] models)
```

:   **Parameters:**: `cidx` -: `tidx` -

---


### setChromName

```
public void setChromName(int cidx,
                         java.lang.String name)
```

:   **Parameters:**: `cidx` -: `name` -

---


### setTraitNames

```
public void setTraitNames(int cidx,
                          java.lang.String[] traits)
```

:   **Parameters:**: `cidx` -: `name` -

---


### setCriterion

```
public void setCriterion(java.lang.String critName)
```

:   Set the criterion name which was used to obtain this model

    :   **Parameters:**: `critName` -

---


### getModel

```
public int getModel(int i,
                    int j)
```

:   **Parameters:**: `i` -: `j` - **Returns:**

---


### getCriterion

```
public java.lang.String getCriterion()
```

:   **Returns:**


---


|  |  |  |
| --- | --- | --- |
| **Overview** \| **Package** \| **Class** \| **Use** \| **Tree** \| **Deprecated** \| **Index** \| **Help** | |  |
| **PREV CLASS**   **NEXT CLASS** | **FRAMES**    **NO FRAMES**     **All Classes** |  |
| SUMMARY: NESTED \| FIELD \| CONSTR \| METHOD | DETAIL: FIELD \| CONSTR \| METHOD |  |


---
